# Study on advance care planning in care dependent community-dwelling older persons in Germany (STADPLAN): protocol of a cluster-randomised controlled trial

**DOI:** 10.1186/s12877-020-01537-4

**Published:** 2020-04-17

**Authors:** Rieke Schnakenberg, Katharina Silies, Almuth Berg, Änne Kirchner, Henriette Langner, Yuliya Chuvayaran, Juliane Köberlein-Neu, Burkhard Haastert, Birgitt Wiese, Gabriele Meyer, Sascha Köpke, Falk Hoffmann

**Affiliations:** 1 Department of Health Services Research, Faculty of Medicine and Health Sciences, Carl von Ossietzky University Oldenburg, Oldenburg, Germany; 2 Institute for Social Medicine and Epidemiology, Nursing Research Unit, University of Lübeck, Lübeck, Germany; 3 Medical Faculty, Institute for Health- and Nursing Science, Martin Luther University Halle-Wittenberg, Halle, Germany; 4 Center for Health Economics and Health Services Research, Schumpeter School of Business and Economics, University of Wuppertal, Wuppertal, Germany; 5 mediStatistica, Neuenrade, Germany; 6 Institute for General Practice, Hannover Medical School, Hannover, Germany

**Keywords:** Advance care planning, Study protocol, Home care setting, Cluster-RCT

## Abstract

**Background:**

In Germany, advance care planning (ACP) was first introduced by law in 2015. However, ACP is still uncommon in Germany and only few people have advance directive forms. This study aims to evaluate an ACP program in care dependent community-dwelling persons, compared to optimised usual care.

**Methods:**

A cluster-randomised controlled trial of 12 months duration will be conducted in 3 German study sites comparing the pretested ACP-counselling offered by trained nurses with a control group receiving optimised usual care. Using external concealed randomisation, 16 home care services each will be included in the intervention and the control group (30 participants per cluster; *n* = 960). Eligibility criteria for patients are: ≥60 years, somehow care dependent, adequate German language skills, assumed life-expectancy of ≥4 weeks, and cognitive ability for participation.

ACP will be delivered by trained nurse facilitators of the respective home care services and communication will include proxy decision-makers. The primary endpoint will be patient activation, assessed by the Patient Activation Measure (PAM-13). Secondary endpoints include ACP-engagement, proportion of prepared advance directives, number and duration of hospitalisations, quality of life as well as depression and anxiety. Further, comprehensive economic and process evaluations will be conducted.

**Discussion:**

STADPLAN is the first study in Germany that assesses an adapted ACP intervention with trained nurses in home care services and the first international study focusing on cost effectiveness of ACP in community-dwelling older persons. The results will help to improve the understanding and communicating of patients’ preferences regarding medical treatment and care and thereby contribute to patients’ autonomy.

**Trial registration:**

German Clinical Trials Register: DRKS00016886 (Date of registration: 04.06.2019).

## BACKGOUND

Worldwide demographic changes lead to increased numbers of care dependent and chronically ill older persons [1, 2]. Functional and cognitive impairment are often accompanied by multimorbidity as well as nursing home admissions and frequent hospital stays [3–5]. Furthermore, care dependency decreases life expectancy [6]. For Germany, the number of care dependent persons is estimated to rise from 3.4 million today [7] to over 4.5 million in 2050 [8], of whom most will be community-dwellers [9].

Shared decision making with respect to nursing and medical decisions as well as improved communication processes between patients and health care professionals are essential elements of high-quality care [10]. In Germany, shared decision making has been included in the Patients’ Rights Act “Patientenrechtegesetz” since 2013.

Therefore, the concept of advance care planning (ACP) has become increasingly important. ACP is a process of discussing in advance a person’s health care preferences and wishes regarding future treatment especially if – due to physical or mental deterioration – the person cannot state his or her wishes anymore [11]. The communication process takes place between individuals and specifically skilled facilitators such as nurses, social workers, or physicians and may also involve relatives.

Persons are encouraged to document their future treatment preferences in advance directives (AD) and update them regularly as health status and preferences might change over time. By these repeated conversations, health care professionals and relatives learn about a person’s priorities, beliefs, values, and choices and therefore are able to arrange treatment accordingly in potential future situations [11]. In this way, ACP extends a persons’ autonomy to a phase in life where he or she becomes incapacitated.

Although the German Advance Directives Act “Patientenverfügungsgesetz” of 2009 confirmed that ADs are binding and the prevalence has been rising during the last years, ACP is still not widely implemented in Germany [12]. Since 2015, the Act to improve Hospice and Palliative Care (“Hospiz- und Palliativgesetz”) stipulates that ACP in German nursing homes is funded by the health insurance funds. However, only few residents, have an AD and most fail to accurately state what should be done when the person acutely becomes incapable of participating in treatment decisions [13, 14]. A patient’s AD may also be disregarded by medical and nursing staff due to insufficient communication.

Therefore, successful ACP programs require a cross-sectoral, multidisciplinary approach, which implies improved communication even among all healthcare providers [15]. Studies from other countries have shown that ACP can reduce distress, depression and anxiety not only in residents but also in significant others and care staff [16, 17]. However, compared to optimised usual care, ACP programs have not been studied solely in community-dwellers [18, 19]. Therefore, no evidence is currently available on the effectiveness of ACP programs implemented by trained nurse facilitators in the home care setting. The cost-effectiveness of ACP is not well understood, although some evidence indicates that ACP is associated with healthcare savings in nursing home residents [20–22].

### Objective

The main focus of this study is to evaluate the effect of an ACP program on patients’ activation regarding healthcare issues, compared to optimised usual care in care dependent community-dwelling older persons.

Further aims are to assess the intervention’s impact on patients’ ACP engagement as well as prepared AD forms, hospitalisations and institutionalisations, anxiety and depression, self-reported health-related quality of life, and mortality. A process evaluation developed according to the MRC framework [23] will be performed to analyse the implementation process, mechanisms of impact and contextual factors that might influence the outcome of the intervention. A health economic evaluation will assess the cost implications of the intervention over 12-months of follow-up.

## Methods

### Study design and setting

**Fig. 1:**
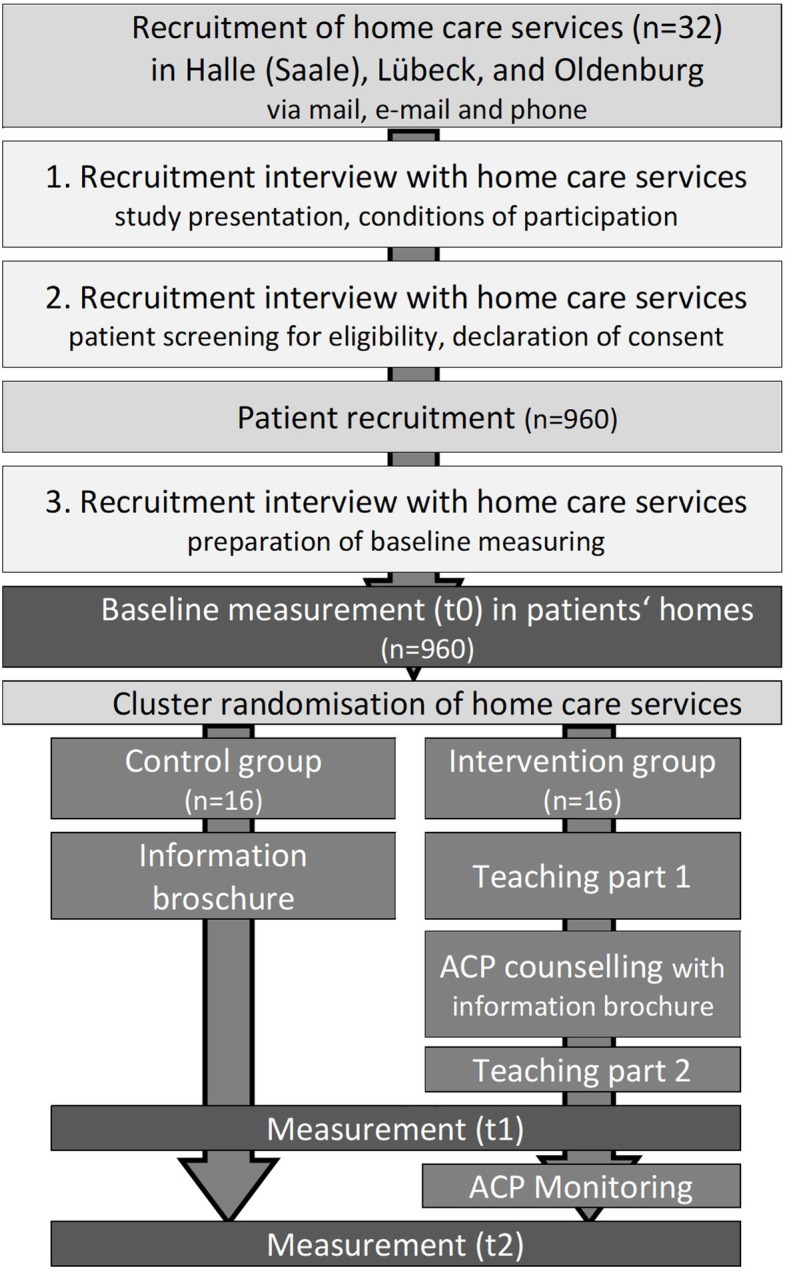
Study flow chart

STADPLAN (STudy on ADvance care PLANning in care dependent community dwelling older persons) is a two-arm cluster randomised controlled trial of 12-months follow-up, conducted to evaluate the effect of an ACP program compared to optimised usual care for people living at home receiving professional home care. Figure 1 summarises the process of enrolment, randomisation, and measurement points during follow-up.

### Preparatory work

The patient-centred ACP programme “Respecting Choices®” [24] was adapted to the home care setting and the German context (GM, AB, HL, ÄK). The intervention components were systematically adapted to the needs of community nursing care in Germany and guided by the Behaviour Change Wheel method [25]. Compared to the “Respecting Choices®” program, the adapted version is less time consuming and more feasible for nurses who will deliver the ACP counselling under “real-life” conditions. The aim is not only to prove effectiveness, but also feasibility and potential for future implementation within existing structures rather than setting up parallel new structures.

The adapted intervention of the STADPLAN study was piloted with four home care services (two each in Halle (Saale) and Oldenburg) in 2018. The pilot study included the whole intervention, the recruitment of home care services and patients, baseline measurements, and parts of the process evaluation up to 3 months after baseline measurement. It helped to optimise the feasibility regarding recruitment, as one result was to minimize the time required for participation. One result was, that the acceptability could be raised by means of decreasing the age from 65 to 60 as inclusion criterion, because the nursing services stated to find it relevant not to exclude the younger clients from information regarding ACP. Moreover, it was helpful to maximise the understanding of used forms like written information and the conversation guide as well as the acceptability of the intervention parts and organisational factors by getting direct feedback from the nurses.

### In- and exclusion criteria for home care services

In total, 32 home care services will be recruited throughout the catchment areas of the three study sites Lübeck, Oldenburg (both north western Germany) and Halle (Saale) (eastern Germany). Each home care service has to care for 70 patients or more and be willing to assign at least two key nurses for the ACP training as well as for the counselling conversations. Minimum qualification of the participating nurses is 3 year professional education (registered nurse) or a bachelor’s degree. Key nurses recruit participating patients and in the intervention group conduct the ACP counselling additionally. Home care services with mainly or solely special alignment, e.g. palliative, paediatric or intensive care will not be considered.

#### In- and exclusion criteria for patients

To be eligible, patients have to be (i) clients of a participating home care service, (ii) aged 60 years or older, (iii) assigned to a care grade of 1 or higher, and (iv) rated into a life-expectancy of at least 4 weeks. Furthermore, (v) adequate German language skills, and (vi) the cognitive ability to follow the intervention and data collection are required. The latter will be assessed using the stepwise approach of the Dementia Screening Scale (DSS), a validated seven-item proxy instrument for nurses where a score of “0” indicates no cognitive impairment and “14” the most severe cognitive impairment [26]. Patients with a total DSS score < 3 will be included in the study whereas those scoring between 3 and 5 will only be eligible if the respective key nurses consider them competent to follow the intervention and to give informed consent. No further exclusion criteria will be applied.

An expected mean of 30 patients are aimed to be included per home care service (*n* = 960 patients).

### Recruitment

A total of 288 home care services are located in the three study sites Oldenburg, Lübeck and Halle (Saale) [27]. Of those 32 (11%) will be recruited. The selected home care services will be invited to take part in the study via postal mail and subsequent telephone calls. This strategy has been successfully applied in previous studies conducted in nursing homes [28, 29] or home care services [30]. The recruitment process will be documented including dates, contact times, and feedback.

Interested home care services have to take part in three separate recruitment interviews provided by study assistants, following the structure presented in Fig. 1 and involving the key nurses, who will thereby be prepared for the standardised patient recruitment. Key nurses will prepare a list of all patients fulfilling the inclusion criteria. This list will be ordered randomly and subsequent patients will be asked for participation until 30 patients will be recruited.

### Randomisation

Randomisation will be carried out on the cluster level (i.e. the home care service) by one investigator (JKN), who is not involved in the recruitment process. Thus, allocation concealment will be guaranteed. Clusters will be allocated 1:1 between intervention and control group. Computer-generated lists will be used with block sizes of two in order to inform the home care services as fast as possible so they can reliably plan time for intervention. Each pair will be allocated simultaneously. Randomisation will be stratified by study site (Oldenburg, Lübeck and Halle (Saale)).

### Intervention and control

The STADPLAN study uses a complex intervention which was established in accordance with the UK Medical Research Council’s (MRC) guidance for developing and evaluating complex interventions [31]. Respecting Choices® was implemented in the United States since 1990 [32] and is conceptually based on the ethical principles of informed consent, best interest and shared decision making [17, 33]. The intervention will be conducted on two levels: (i) the home care service and (ii) the patient level (see Table 1).

**Table 1:** Intervention elements of the STADPLAN study

Intervention group	Control group (optimised usual care)
**Home care service level**	
**2-day training for participating nurses** Divided into 7 modules: M1: Introduction of the STADPLAN study M2: Introduction of the topic ACP M3: Practical exercise of the counselling conversations, extensive practise of the guided conversations with partners using different health situations/cases M4: Facilitator’s tasks and schedule in the course of the study M5: Reflexion on conversation experiences M6: Special practical training of difficult conversational situations, refresher of knowledge on ACP M7: Feedback and closing of the training	**–**
**Patient level**	
**ACP counselling (divided into 2 parts)** **Part 1**: Information on the project, ACP, aim of the conversations, information on the tasks and features of the surrogate/ representative, information on the written living will, introduction of the written information brochure, preparation of the next conversation: topic and goal, presence of a representative, **Part 2**: Repeating information on project, ACP and aim of the conversation, introduction of the following topics: attitudes, preferences and values of the participant, reflection on the use of the additional written information and integration of notes, clarification: further conversations requested?	**–**
**Written information on ACP:** Information brochure of about 60 pages containing: • Introduction to ACP, surrogate decision making and advance directive documents • Presentation of critical health scenarios along with incapacity • Glossary of medical and legal terms, contact information on local consultancies	**Written information on ACP:** Brochure of about 15 pages containing: • Introduction to ACP, surrogate decision making and advance directive documents (condensed) • Presentation of critical health scenarios along with incapacity (condensed) • Glossary of medical and legal terms, contact information on local consultancies

*ACP* advance care planning, *BEVA* trained nurse facilitator

#### Intervention group

In a 2-days workshop, key nurses from the respective home care services will receive ACP training and be prepared for their role as facilitator (short German version: “BEVA”). This workshop will provide an introduction to ACP and inform about the possibilities for ADs. Further, BEVAs will train counselling conversations using the conversation guide developed in the STADPLAN study. Finally, conversation experiences and problems, as well as coping strategies will be discussed.

On the patient level, the intervention will include two components which consist of (i) a formal ACP counselling provided by the BEVA and (ii) a written information brochure. The first component, the ACP counselling, is divided into two counselling meetings at the patient’s home. The first conversation lasts about 30 min and follows the developed and pre-tested guideline (Table 1). The written information brochure will be delivered and explained during this first conversation. In a structured approach, this brochure encourages the discussion of health care preferences and wishes regarding future treatment in case a person cannot state his or her opinion anymore. It further includes a glossary of medical and legal terms as well as contact information on local consultancies. The second conversation is also based on a semi-structured guideline and lasts about 60 min. The patient’s proxy decision maker or another person of trust, if available, will be invited to take part.

Reasons for discontinuing intervention are withdrawing consent, death, or moving to a nursing home.

The intervention’s primary aim is to promote patients’ awareness of ACP and their activation with respect to future treatment preferences. It also supports the conversation between patients and their proxy about wishes and treatment aims and motivates to this process to be able to take into account future changes or modifications of the patients’ wishes. The completion of AD forms is not part of the intervention.

#### Control group

Patients in the control group will receive optimised usual care, i.e., provision of written information on ACP (an abbreviated version of the information brochure) at the beginning of the study.

After completion of the data collection, interested nurses of the home care services in the control group will be offered a 1-day workshop on ACP.

### Primary outcome

The primary outcome is patient activation, assessed by the Patient Activation Measure (PAM-13-D) [34] at 12 months. The PAM-13 is a valid and reliable instrument which measures the degree to which individuals take an active role in managing their own health, the corresponding health care and its consequences, and the extent to which they feel competent to fulfil that role [35]. The German version (PAM-13-D) has shown to be a reliable and valid measure of patient activation [36, 37]. Each item can be answered with one of four possible response options, which range from “disagree strongly” [1] to “agree strongly” [4]. One item (no. 4) has a fifth response option, namely “I do not take medications”. Raw scores are added up (range 0–100) with higher scores indicating more participation. The PAM-13-D will be assessed via face-to-face interviews by trained study assistants who are blinded to the group allocation of clusters.

### Secondary outcomes

The secondary outcomes are the proportion of participants with ADs, self-reported health related quality of life (VR-12 with 12 items) [38, 39], anxiety and depression (HADS with 14 items) [40], number of hospitalisations, proportion of participants being institutionalized, mortality, and ACP engagement with four items on readiness to pass the ACP process [41] (Table 2). Data measurement will be conducted at baseline (t0), after 6 months (t1) and after 12 months (t2). Baseline data will include age, sex, family situation, amount of health care services used, selected comorbidities, hearing capacity, vision, mobility, and functional status. Most of these data will be assessed in face-to-face interviews with the patients at their homes. Data routinely documented by the respective home care services will be collected during visits at the home care service.

**Table 2:** Study outcomes/variables, measurement tools and data collection schedule

Outcome/ variable	Measured by	Validated version*	Baseline	6 months (t1)	12 months (t2)	Assessed by
Patient activation (primary outcome)	Patient activation Measure (PAM-13) [42]	German version [36]	✓	✓	✓	RA – at home visit
ACP behaviour	Patient engagement survey [41]	English version [41]	✓		✓	RA – at home visit
Anxiety and depression	Hospital anxiety and depression scale (HADS) [40]	German version [43]	✓		✓	RA – at home visit
Self-reported, health-related quality of life	Veterans Health Administration (VR-12) [38, 39]	German version [44]	✓		✓	RA – at home visit
Deaths, hospitalization, institutionalisation	Own questions	n.a.	✓	✓	✓	RA – at home care service & home visit
Proxy/Family involvement**	Audit of ACP discussion	n.a.	✓			Nurse facilitator
Sociodemographic & clinical data, hearing capacity by RAI-NH [45] & vision	Own questions	n.a.	✓			RA- at home care service & home visit
GPs involvement	Own questions	n.a.	✓	✓	✓	
Physician visits, formal & informal care	Own questions	n.a.	✓		✓	RA – at home visit
Healthcare preferences	Hypothetical scenarios	Adapted German version [46]***	✓		✓	RA – at home visit

*all instruments were pretested in a German version in the pilot study; **intervention group only; *** adapted version of the Life-Support Preferences Questionnaires and Emanuel Medical Directive, which will be validated in the STADPLAN study within a limited subgroup (*n* = 120); *RA* Research assistant, *RAI-NH* Resident Assessment Instrument for Nursing Homes

### Process evaluation

The comprehensive process evaluation follows the UK MRC guidance for the process evaluation of complex interventions [47], aiming to identify the context of the intervention delivery, assess implementation and intervention fidelity, identify mechanisms of impact and interpret outcomes in the light of these identified processes. Thus, observed effects can be interpreted in the light of how the intervention actually worked. Methods comprised are qualitative interviews, focus groups, written questionnaires, and extensive documentation of the ongoing research process, based on a pre-specified logic model. A more comprehensive protocol for the process evaluation will be submitted later.

### Health economic evaluation

In consideration of O’Hanlons’ framework on economic evaluations of ACP [48], the objective of the health economic evaluation is to estimate implementation and intervention costs during the study period. Cluster adjustment will be performed. Further, cost implications of the ACP intervention will be explored.

The estimation of (i) implementation und intervention costs will be performed from the perspective of the home care services (on organizational level) as well as the German social insurance system (on patient level). Cost implications of the intervention (ii) during the 12-month follow-up, in particular inpatient care costs (hospital as well as short-term nursing home), expenses for rehabilitation services and medical devices, will be determined from the perspective of the German social insurance system (on patient level). Cost implications of the intervention, which (iii) could be expected in following years and therewith after the end of our study will be explored by using hypothetic scenarios (on patient level). The scenarios were developed according to international literature on ACP [48–51] and pre-tested during the pilot phase prior the STADPLAN study.

For (i) and (ii), costs for implementation- and intervention-related components will be collected during the study. In a first step, resource use associated with ACP implementation and intervention (e.g. ACP facilitators, qualified nurses, time and material) as well as related health care resource use will be assessed during the study. In a second step, resource use will be multiplied by unit costs.

The scenarios, which are used for the calculation of point (iii), describe the goals of care chosen by a random subgroup of 120 study participants on cluster level at baseline and at the end of the study for various hypothetic health events. These goals of care will be transformed into costs in three steps: first, we will identify the healthcare resources required to treat the hypothetic health event; second, the amount of resources has to be determined and third, each resource will be multiplied by unit costs. The difference in treatment costs resulting of the decision made at baseline and at the end of the study will be compared between the intervention and the control group.

### Sample size

To detect an effect size of 0.35 (Cohens d), which corresponds to a medium effect, with respect to the primary endpoint after 12 months between the intervention and the control group with 90% power (β = 0.10) using a two-sided significance level of 5% (α = 0.05) based on the t-test, a non-cluster study setting would require a total of 173 patients per group. Taking cluster sampling into account [52], we used an intra-cluster correlation coefficient (ICC) of 0.05 in accordance with a study on the effectiveness of ACP in the Netherlands of Korfage et al. [20]. Assuming an average cluster size of 30 participants per home care service, the design factor is 2.45 and 15 clusters are required per group. Estimating that two home care services might drop out [21], 32 clusters with a total of 960 participants will be included in the study.

### Data analysis

Data analyses will be conducted by a blinded biometrician (BH), who does not know, which group is intervention or control, following Good Clinical Practice (GCP) standards. All analyses will be cluster-adjusted and follow the intention-to-treat principle.

Baseline characteristics will be displayed descriptively. The primary outcome (PAM-13-D) will be compared between intervention and control group using a mixed model (α = 0.05, two-sided) and adjusted for fixed (baseline values including initial value of PAM-13-D) and random (cluster) effects. For patients who early terminated the study, last observations (information at the last point of measurement) will be carried forward (LOCF imputation). For sensitivity, the same analysis will be performed as complete case analysis without LOCF. Furthermore, a more detailed analysis comparing the longitudinal course of the primary outcome in the interim time points after 6 and 12 months without imputation will be investigated using a mixed model with the intervention and initial PAM-13-D values as fixed effects, clusters as a random effect, and using covariance patterns to adjust for repeated measurement.

Mixed models will also be used for all secondary endpoints, taking into account cluster effects or repeated measurements. Survival analyses will be conducted using stratified Kaplan-Meier curves and Cox regression. Subgroup analyses will be conducted stratified by age and sex.

Economic analyses on patient level will include individual total costs over 12 months. Differences between intervention and control group will be estimated using Gamma regression, again accounting for cluster effects. Adjustment is planned for prematurely dropped out. On organizational level (implementation and intervention costs from the perspective of home care services) descriptive analyses are will be performed.

For the process evaluation, qualitative data (interviews and focus groups) will be summarised and narratively described [53] supported by MAXQDA. Further, quantitative data (questionnaires with closed- and open-ended questions) will be analysed using descriptive statistics.

### Data management

All data will be collected by blinded study assistants at each study site using standardised Case Report Forms (CRFs). Afterwards, the CRFs will be digitalised using the data management system secuTrial®. Extensive plausibility checks and data validation will be conducted by the trained researcher (BW), who has also developed and proven the database following GCP rules. Data of drop outs will be analysed until drop out date (intention-to-treat), except the withdrawing consent goes along with the wish to delete all data. All personalised contact data and key lists remain under lock at the respective study site so that confidentiality will be preserved.

### Trial status

Home care service recruitment started in April 2019 and patient recruitment was completed in January 2020. The baseline was also completed in January 2020 and the 12-month follow-up will accordingly be completed in January 2021. Analyses will be completed in March 2021.

### Patient and public involvement

Patients and public were not involved in the definition of the research questions or outcome measures. However, patients and their relatives were involved in the pilot study, as feasibility and acceptability were tested also from their point of view. Additionally, one of the largest organisations of relatives of care dependent persons in Germany supports the STADPLAN study and is represented on the advisory board representing medical, statistical, medical law, ethics and patient advocacy expertise. Furthermore, pilot study results were presented and discussed on international conferences before finalizing the concept for the main study.

## Discussion

The STADPLAN study takes place given the fact that most of the recent international publications on ACP interventions were conducted or are actually running in the primary care setting [54–56] or in the hospital setting [57–59] after a time of investigating ACP mainly in the nursing home setting [60]. However, more evidence on the effectiveness of ACP-interventions in different settings is needed, also taking into account the structures of respective healthcare systems. STADPLAN is the first study in Germany that assesses an adapted ACP intervention in the home care service setting using trained nurses as facilitators. Furthermore, it is the first study internationally that focuses on the effectiveness and costs of ACP in community-dwelling older persons. Results will help to improve understanding and communicating patients’ will regarding future medical treatment and care, and thereby contribute to patients´ autonomy at the end of life.

### Limitations

As the ACP intervention will be provided only in German and only to patients being cognitively able to follow the intervention, patients with inadequate German language skills or with dementia will be excluded. As the study participation will take at least 40 h per home care service at the intervention group as well as the target population is a vulnerable older group, it cannot be excluded, that higher drop-out rates will occur. Drop-outs might cause a potential bias. We plan analyses using imputation by LOCF without multiple imputation, and additionally an analysis of the complete time course to discuss a possible bias of the result form drop-outs.

### Strengths

The study has several strengths. We will assess the effectiveness of ACP for the first time in the home care setting. Further, the cluster-randomised design will provide a high level of evidence on the question whether patient activation may be increased by our intervention. Apart from the experimental interventions, control and intervention group clusters will be treated equally in order to prevent performance bias. As successfully performed in previous studies [29, 61, 62], we will spend a lot of effort to avoid cluster drop-outs (e.g. by regularly telephone calls with the BEVAs and various offers for support), which could lead to cluster imbalance and pronounced lost to follow up. Additionally, the study duration of 12 months provides the possibility to assess long-term effects.

A further strength of our study is the extensive process evaluation since changing attitudes or behaviours in complex interventions pose the challenge of determining how contextual components of participants and home care services interact and influence the intended outcome.

## Data Availability

Not applicable.

## Abbreviations

ACP: Advance Care Planning
AD: Advance directive
BEVAs: Trained nurse facilitator
CRF: Case report form
DSS: Dementia Screening Scale
GP: General practitioner
HADS: Hospital Anxiety and Depression Scale
ICC: Intra-cluster correlation coefficient
LOCF: Last observation carried forward
NH: Nursing home
PAM: Patient Activation Measure
RA: Research assistant
STADPLAN: Study on advanced care planning in care dependent community-dwelling older persons
